# Supplementary material for: The impact of lockdown enforcement during the SARSCoV-2 pandemic on the timing of presentation and early outcomes of patients with ST-elevation myocardial infarction
Source: PLoS One. 2020 Oct 23;15(10):e0241149. doi: 10.1371/journal.pone.0241149 (PMC7584161; doi:10.1371/journal.pone.0241149)
Supplement: S1 Table — (DOCX) [file pone.0241149.s001.docx]

S1 Table. Weekly confirmed new SARS-CoV-2 Cases in Israel during Lockdown.

|  | New cases | Incidence per 1M population |
| --- | --- | --- |
| March 16th | 1,053 | 114 |
| March 23rd | 3,166 | 344 |
| March 30th | 4,307 | 468 |
| April 6th | 2,768 | 300 |
| April 13th | 2,310 | 251 |
| April 20th | 1,574 | 171 |
| April 27th | 712 | 77 |
| May 4th | 272 | 29 |

Data source: https://covid19.who.int/region/euro/country/il
